# Development of a Prognostic App (iCanPredict) to Predict Survival for Chinese Women With Breast Cancer: Retrospective Study

**DOI:** 10.2196/35768

**Published:** 2022-03-09

**Authors:** Zhuo Ma, Sijia Huang, Xiaoqing Wu, Yinying Huang, Sally Wai-Chi Chan, Yilan Lin, Xujuan Zheng, Jiemin Zhu

**Affiliations:** 1 Department of Nursing School of Medicine Xiamen University Xiamen China; 2 Department of Chronic Non-infectious Diseases and Endemic Diseases Control Xiamen Center for Disease Control and Prevention Xiamen China; 3 Department of Nursing, Women and Children's Hospital School of Medicine Xiamen University Xiamen China; 4 President Office Tung Wah College Hong Kong China; 5 School of Nursing Health Science Centre Shenzhen University Shenzhen China

**Keywords:** app, breast cancer, survival prediction model, iCanPredict

## Abstract

**Background:**

Accurate prediction of survival is crucial for both physicians and women with breast cancer to enable clinical decision making on appropriate treatments. The currently available survival prediction tools were developed based on demographic and clinical data obtained from specific populations and may underestimate or overestimate the survival of women with breast cancer in China.

**Objective:**

This study aims to develop and validate a prognostic app to predict the overall survival of women with breast cancer in China.

**Methods:**

Nine-year (January 2009-December 2017) clinical data of women with breast cancer who received surgery and adjuvant therapy from 2 hospitals in Xiamen were collected and matched against the death data from the Xiamen Center of Disease Control and Prevention. All samples were randomly divided (7:3 ratio) into a training set for model construction and a test set for model external validation. Multivariable Cox regression analysis was used to construct a survival prediction model. The model performance was evaluated by receiver operating characteristic (ROC) curve and Brier score. Finally, by running the survival prediction model in the app background thread, the prognostic app, called iCanPredict, was developed for women with breast cancer in China.

**Results:**

A total of 1592 samples were included for data analysis. The training set comprised 1114 individuals and the test set comprised 478 individuals. Age at diagnosis, clinical stage, molecular classification, operative type, axillary lymph node dissection, chemotherapy, and endocrine therapy were incorporated into the model, where age at diagnosis (hazard ratio [HR] 1.031, 95% CI 1.011-1.051; *P*=.002), clinical stage (HR 3.044, 95% CI 2.347-3.928; *P*<.001), and endocrine therapy (HR 0.592, 95% CI 0.384-0.914; *P*=.02) significantly influenced the survival of women with breast cancer. The operative type (*P*=.81) and the other 4 variables (molecular classification [*P*=.91], breast reconstruction [*P*=.36], axillary lymph node dissection [*P*=.32], and chemotherapy [*P*=.84]) were not significant. The ROC curve of the training set showed that the model exhibited good discrimination for predicting 1- (area under the curve [AUC] 0.802, 95% CI 0.713-0.892), 5- (AUC 0.813, 95% CI 0.760-0.865), and 10-year (AUC 0.740, 95% CI 0.672-0.808) overall survival. The Brier scores at 1, 5, and 10 years after diagnosis were 0.005, 0.055, and 0.103 in the training set, respectively, and were less than 0.25, indicating good predictive ability. The test set externally validated model discrimination and calibration. In the iCanPredict app, when physicians or women input women’s clinical information and their choice of surgery and adjuvant therapy, the corresponding 10-year survival prediction will be presented.

**Conclusions:**

This survival prediction model provided good model discrimination and calibration. iCanPredict is the first tool of its kind in China to provide survival predictions to women with breast cancer. iCanPredict will increase women’s awareness of the similar survival rate of different surgeries and the importance of adherence to endocrine therapy, ultimately helping women to make informed decisions regarding treatment for breast cancer.

## Introduction

Accurate prediction of survival will help both physicians and women with breast cancer to determine the type of surgery and the adjuvant therapy that are beneficial for these women [1]. Physicians usually make survival predictions and adjuvant therapy formulations according to the clinical characteristics of women with breast cancer, such as age, tumor size, clinical stage, hormone receptor status, and molecular characteristics, in addition to their own clinical experience [1-3]. The predicted adjuvant benefit is achieved by calculating the percentage improvement in the predicted survival rate after adjuvant treatment [1].

Currently, some survival prediction tools are widely used, such as PREDICT [1,4] and Adjuvant! Online [5-9]. PREDICT was developed based on clinical data of women with breast cancer collected from East Anglia (UK) between 1999 and 2003 [1,4], and Adjuvant! Online was developed based on data at the Netherlands Cancer Institute from 1987 to 1998 [5-9]. Studies have shown that such prediction tools underestimated or overestimated the survival rate of women with breast cancer, especially in Asia [10-12]. Differences in access to health care and the quality of existing health care among countries are possible reasons [13].

In China, the practice of surgery for breast cancer markedly differs from that in Western countries. A national survey reported that breast-conserving surgery accounted for 22% of surgeries in China [14], in contrast to 64.5% in the United States [15], despite the similar survival rates of breast-conserving surgery and mastectomy for women with early stage breast cancer [16,17]. Furthermore, some recent studies have shown that breast-conserving surgery has higher breast cancer–specific and overall survival rates than mastectomy [18]. The insufficient and unbalanced medical resources, information disparity between physicians and women with breast cancer, distrust of breast-conserving surgery by some physicians and women, and fear of recurrence are important reasons for the low rate of breast-conserving surgery in China [14]. In addition, for women with breast cancer in China who had received adjuvant endocrine therapy, the nonpersistence rates ranged from 5.9% to 22.6% in years 1-5, and the rate of low compliance ranged from 2.1% to 11.2% [19]. The low compliance with endocrine therapy may be partially explained by inadequate information and social support [19-23]. Therefore, demonstrating the similar survival rate of different surgeries and the efficacy of adherence to endocrine therapy may help physicians and women with breast cancer make better clinical decisions.

In 2020, the popularity of mobile phones in China exceeded 1 per person [24], and 920 million people could surf the internet using mobile phones [25]. Further, WeChat, a popular mobile phone platform, has over 1 billion monthly active users in China [26]. The Mini app, one of the functionalities of WeChat, provides an easily accessible platform to present the Chinese survival prediction model for women with breast cancer.

To the best of our knowledge, no prognostic app has been designed and applied to women with breast cancer in China. Such survival prediction tools would be beneficial to help physicians and women with breast cancer obtain and understand the survival rate of different surgeries and adjuvant therapy, thereby enabling better clinical decision making. The aim of this study is to develop and validate a prognostic app to predict the overall survival of women with breast cancer in China.

## Methods

### Study Design

A retrospective design was applied. All samples were randomly divided (7:3 ratio) into a training set for model construction and internal validation, and a test set for model external validation [27].

### Study Setting and Population

Clinical data collection was conducted at 2 hospitals in Xiamen, a tertiary hospital and a hospital for women and children health. Data on death were collected from the Xiamen Center of Disease Control and Prevention (CDC), which retains all death records for Xiamen residents.

The inclusion criteria were women with breast cancer (1) who received treatment including surgery and adjuvant therapy in these 2 hospitals from January 1, 2009, to December 31, 2017; and (2) who were Xiamen residents. Women who lacked surgical and treatment information were excluded from the analysis.

### Sample Size Calculation

The pmsampsize package for Stata and R written by Riley et al [28] was used to calculate the sample size. The 10-year mortality rate in women with breast cancer was 0.214, and the median follow-up was 11.7 years [6]. To target a margin of error in the estimate of death risk of ≤0.05, a total of 680 samples were needed with Cox-Snell R^2^ statistic of 0.1 [28], assumed outcome event rate of 0.214, follow-up of 11.7 years [6], timepoint of interest for 10 years’ prediction, and 8 parameters (variables).

### Procedure

The researchers collected clinical data of all patients with breast cancer who received treatment at 2 participating hospitals from January 2009 to December 2017. Further, the population information on death for Xiamen residents from January 2009 to December 2020 was obtained from the Xiamen Center CDC. The citizen ID number was used as the basis for matching. If the match was successful, the death dates of those women were recorded. If not successful, women with breast cancer were assumed to be alive. Finally, a data set was developed for model construction and validation.

### Ethical Consideration

Ethical approvals were obtained from Research Ethics Committees at School of Medicine, Xiamen University (number XDYX2019008), First Hospital Affiliated to Xiamen University (number XMDY-2020-019), and Women and Children’s Hospital affiliated to Xiamen University (number KY-2019-058). To protect the privacy of women with breast cancer, data collection, entry, and processing were carried out by 2 professional researchers. During the use of iCanPredict, the users’ input information can only be accessed by authorized researchers, who needed to log-in to the iCanPredict website background thread with the protected account number and passport. Accordingly, unauthorized personnel was prohibited from viewing and using the clinical data and users’ input information.

### Data Collected

Outcomes data were obtained from the medical records (January 2009-December 2017) of the 2 participating hospitals including age at diagnosis, clinical stage (stage 0-IV), molecular classification (luminal A, luminal B, Her-2 overexpression, basal-like), operative type (breast conservative surgery, mastectomy), breast reconstruction, axillary lymph node dissection, chemotherapy, and endocrine therapy. These 2 participating hospitals used the TNM clinical stages from the American Joint Committee on Cancer, which are determined by tumor size (T), lymph node metastasis (N), and distant metastasis (M) [29].

Xiamen residents’ death information from January 1, 2009, to December 15, 2020, was collected from the Xiamen Center CDC. Women’s citizen ID number was retrieved from the hospital to link women’s medical records to Xiamen residents’ death information at the Xiamen Center CDC.

### Outcomes

The 5-year (or 10-year) mortality was defined as the number of deaths divided by the total number of breast cancer cases from the diagnosis to 5-year (or 10-year) follow-up. The outcome event was the death of women with breast cancer from January 1, 2009, to December 15, 2020. The survival of women with breast cancer at the end of follow-up was considered as a truncated event [30].

### Data Analysis

Data analysis was performed using SPSS version 25.0 (IBM) [31] and R version 4.0.4 (R Foundation). Demographic data were summarized using descriptive statistics. Because all variables satisfied the proportional hazards (PH) assumption, multivariate Cox regression analysis was used to develop a PH regression model for time-to-event data (eg, happening of death) and produce an equation to predict an individual’s outcome risk on the condition of her values of multiple predictors [28].

### Model Construction

The training set was used for model construction. On the basis of the hazard ratio (HR) of each prognosis factor estimated by multivariable Cox regression, the survival prediction model was expressed as



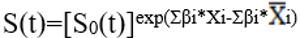



where S(t) indicates the predicted survival rate of women with breast cancer after treatment, S_0_(t) represents the baseline survival rate of women with breast cancer, β is the coefficient for each prognostic factor in the multivariable Cox regression, X is the value of the prognosis factor, and 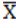 is the mean of the prognosis factor.

### Validation

The training set was analyzed for model internal validation and the test set was analyzed for model external validation. Model discrimination was evaluated using the receiver operating characteristic (ROC) curve. In general, the larger the area under the curve (AUC), the higher the model discrimination. Further, an AUC greater than 0.7 indicates that the model has a certain distinctive ability. Model calibration was based on the Brier score; the lower the Brier score, the higher the calibration degree. When the Brier score was less than 0.25, the model could be predicted.

### Design of a Prognostic App to Predict Survival

The research team comprised researchers, statisticians, breast cancer specialists, and app specialists at Xiamen University, the participating hospitals, and Tung Wah College of Hong Kong. The Xiamen Quanwu Information Service Company (Xiamen, People’s Republic of China) undertook the technical development and maintenance of the iCanPredict app.

iCanPredict is mainly divided into a website background thread (back end) and the user side (front end). The website background thread was developed to approve the users’ applications for registration, run the survival prediction model on the basis of the users’ input information, and track the app usage frequency and duration. Only researchers are able to access the website background thread with protected account number and passport. The users’ side was developed to interact with users and present the 10-year survival rate for women with breast cancer.

According to the Technology Acceptance Model proposed by Davis [32], perceived ease of use and perceived usefulness are 2 decisive variables of the user’s reception of the information system. Perceived ease of use refers to the degree of difficulty a user perceives when using an information system [33]. Perceived usefulness refers to the fact that users can obtain certain values when using an information system [33]. Perceived ease of use affects perceived usefulness and further affects users’ actual use behavior by affecting their attitudes and behaviors [33]. To promote ease of use, the iCanPredict app was incorporated into the functionality of WeChat, the most popular mobile phone platform in China. Prior to implementing the iCanPredict app, the perceived ease of use and perceived usefulness were tested by women with breast cancer and surgeons.

## Results

### Participants’ Demographic and Clinic Characteristics

The clinical data of 1686 participants were collected, of which 97 lacked treatment information, leaving data on 1592 participants for model construction and validation. The training set comprised 1114 individuals and the test set comprised 478 individuals. By December 15, 2020, 147/1592 participants died (9.23%) and 1445/1592 (90.77%) were still alive. In this study, the mean follow-up was 6.38 years, ranging from 0.32 to 12.35 years. The mean age at diagnosis was 49.92 (SD 11.59) years, and most participants were diagnosed with stage II breast cancer (733/1592, 46.04%). Luminal B-type (1059/1592, 66.52%) was the most common molecular classification. Only 95/1592 participants (5.97%) underwent breast-conserving surgery, while 1497/1592 (94.03%) underwent mastectomy. A total of 453/1497 participants underwent breast reconstruction, accounting for 30.26% of the participants who underwent mastectomy and 28.45% (453/1592) of all participants. Most participants underwent axillary lymph node dissection (1143/1592, 71.80%), received adjuvant chemotherapy (1305/1592, 81.97%), and received adjuvant endocrine therapy (1007/1592, 63.25%). The demographic and clinical characteristics of the participants in the training and test sets are presented in Table 1.

**Table 1:** Demographic and clinical characteristics of participants in the training set and the test set.^a^

Characteristics		Total (n=1592)	Training set (n=1114)	Test set (n=478)
Deaths, n (%)		147 (9.23)	103 (9.25)	44 (9.21)
Patients alive, n (%)		1445 (90.77)	1011 (90.75)	434 (90.79)
Follow-up (years), mean (SD)		6.38 (2.68)	6.40 (2.68)	6.32 (2.68)
Age at diagnosis (years), mean (SD)		49.92 (11.59)	49.68 (11.48)	50.50 (11.82)
**Clinical stage, n (%)**				
	0	26 (1.63)	21 (1.89)	5 (1.05)
	I	524 (32.91)	363 (32.59)	161 (33.68)
	II	733 (46.04)	508 (45.60)	225 (47.07)
	III	286 (17.96)	204 (18.31)	82 (17.15)
	IV	23 (1.44)	18 (1.62)	5 (1.05)
**Molecular classification, n (%)**				
	Luminal A	215 (13.51)	160 (14.36)	55 (11.51)
	Luminal B	1059 (66.52)	723 (64.90)	336 (70.29)
	HER-2 (+)	232 (14.57)	168 (15.08)	64 (13.39)
	Basal like	86 (5.40)	63 (5.66)	23 (4.81)
**Operative type, n (%)**				
	Breast-conserving surgery	95 (5.97)	68 (6.10)	27 (5.65)
	Mastectomy	1497 (94.03)	1046 (93.90)	451 (94.35)
**Breast reconstruction, n (%)**				
	Yes	453 (28.45)	321 (28.82)	132 (27.62)
	No	1139 (71.55)	793 (71.18)	346 (72.38)
**Axillary lymph node dissection, n (%)**				
	Yes	1143 (71.80)	810 (72.71)	333 (69.67)
	No	449 (28.20)	304 (27.29)	145 (30.33)
**Chemotherapy, n (%)**				
	Yes	1305 (81.97)	919 (82.50)	386 (80.75)
	No	287 (18.03)	195 (17.50)	92 (19.25)
**Endocrine therapy, n (%)**				
	Yes	1007 (63.25)	707 (63.46)	300 (62.76)
	No	585 (36.75)	407 (36.54)	178 (37.24)

^a^Percentage=number/group total number.

### Initial Model Fit

The training set was used to fit the initial model. The graph based on the standardized Schoenfeld residual method and the –ln(–ln[survival]) test indicated that all 8 variables met the PH assumption. A survival prediction model was constructed for women with breast cancer (Table 2). Age at diagnosis (*P*=.002), clinical stage (*P*<.001), and endocrine therapy (*P*=.02) were identified as significant prognostic factors. The risk of death increased by 0.031 times per year with an increase in age at diagnosis for women with breast cancer (HR 1.031, 95% CI 1.011-1.051). When the clinical stage increased, the risk of death in women increased by 2.044 times per stage (HR 3.044, 95% CI 2.347-3.928). Women with breast cancer receiving endocrine therapy had a 0.407 (1–0.592) times lower risk of death than those that did not receive endocrine therapy (HR 0.592, 95% CI 0.384-0.914). The other 5 variables, including molecular classification (*P*=.91), operative type (*P*=.81), breast reconstruction (*P*=.36), axillary lymph node dissection (*P*=.32), and chemotherapy (*P*=.84), were not significant.

**Table 2:** Hazard ratios and model coefficients for prognostic factors included in the models (n=1114).

Prognostic factors	β	Standard error	Hazard ratio (95% CI)	*P* value
Age at diagnosis	0.031	0.01	1.031 (1.011-1.051)	.002
Clinical stage	1.113	0.133	3.044 (2.347-3.928)	<.001
Molecular classification	0.016	0.145	1.017 (0.765-1.351)	.91
Operative type	0.127	0.52	1.136 (0.410-3.145)	.81
Breast reconstruction	–0.318	0.343	0.728 (0.372-1.426)	.36
Axillary lymph node dissection	0.42	0.422	1.521 (0.665-3.478)	.32
Chemotherapy	–0.067	0.34	0.935 (0.480-1.821)	.84
Endocrine therapy	–0.524	0.222	0.592 (0.384-0.914)	.02

### Validation Results

**Figure 1:**
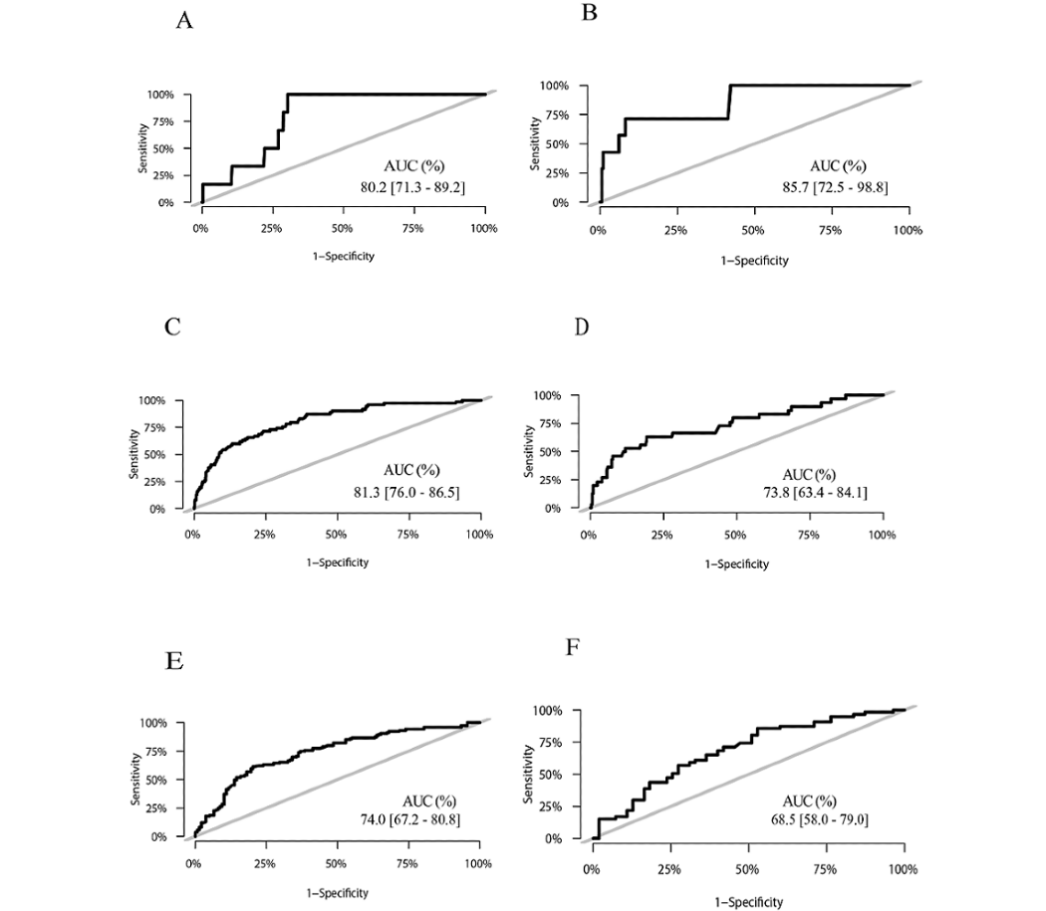
Receiver operator characteristic (ROC) curves for breast cancer overall survival rates. (A) Training set at 1 year; (B) test set at 1 year; (C) training set at 5 years; (D) test set at 5 years; (E) training set at 10 years; (F) test set at 10 years.

All statistically significant and nonsignificant factors are included in the prediction model because all factors are clinically significant. Further, the prediction model including nonsignificant factors presented model discrimination and calibration similar to those presented by the prediction model excluding nonsignificant factors. The AUC and Brier score of the training and test sets are presented in Tables 3 and 4, respectively. For internal validation with the training set, the ROC curve of the training set indicated that the model exhibited good discrimination for predicting 1- (AUC 0.802, 95% CI 0.713-0.892), 5- (AUC 0.813, 95% CI 0.760-0.865), and 10-year (AUC 0.740, 95% CI 0.672-0.808) overall survival (Figure 1A,C,E). The Brier scores at 1, 5, and 10 years after diagnosis were 0.005, 0.055, and 0.103, respectively. For external validation with the test set, except that the AUC at 10 years after diagnosis was 0.685, the AUC values 1 and 5 years after diagnosis were greater than 0.7, indicating that the model has a certain distinctive ability (Figure 1B,D,F). The Brier scores 1, 5, and 10 years after diagnosis were less than 0.25, indicating that the model calibration was also good. Therefore, the prediction model could be used to predict survival.

**Table 3:** Internal validation and external validation: model discrimination (AUC^a^) at 1, 5, and 10 years after diagnosis.

Year	Internal validation		External validation	
	AUC	95% CI	AUC	95% CI
1	0.802	0.713-0.892	0.857	0.725-0.988
5	0.813	0.760-0.865	0.738	0.634-0.841
10	0.740	0.672-0.808	0.685	0.580-0.790

^a^AUC: area under the curve.

**Table 4:** Internal validation and external validation: calibration (Brier) at 1, 5, and 10 years after diagnosis.

Year	Internal validation		External validation	
	Brier score	95% CI	Brier score	95% CI
1	0.005	0.001-0.010	0.014	0.004-0.025
5	0.055	0.043-0.067	0.057	0.038-0.075
10	0.103	0.083-0.124	0.120	0.084-0.156

### Development of Preliminary Survival Prediction for Breast Cancer Survivors

The development of iCanPredict was based on a uniAPP open-source free framework with separation technology for the back end and the front end. The back end was developed using the Java language 8 environment, IntelliJ IDEA for code development, and Maven for service dependency management. The front end utilized an Ant-design-VUE component for page interaction construction under the VUE framework. Each function in the iCanPredict app was tested multiple times to ensure usability and stability.

A QR code was generated for the users to scan with their own WeChat account. After downloading the iCanPredict app into their WeChat platform, the users need apply for registration. The researcher will approve the users’ application from the website background thread. Each user’s mobile phone number is set up as the unique username and an automated passport is sent to the user’s mobile phone (changeable later). The users do not need to pay for the access to the iCanPredict app.

**Figure 2:**
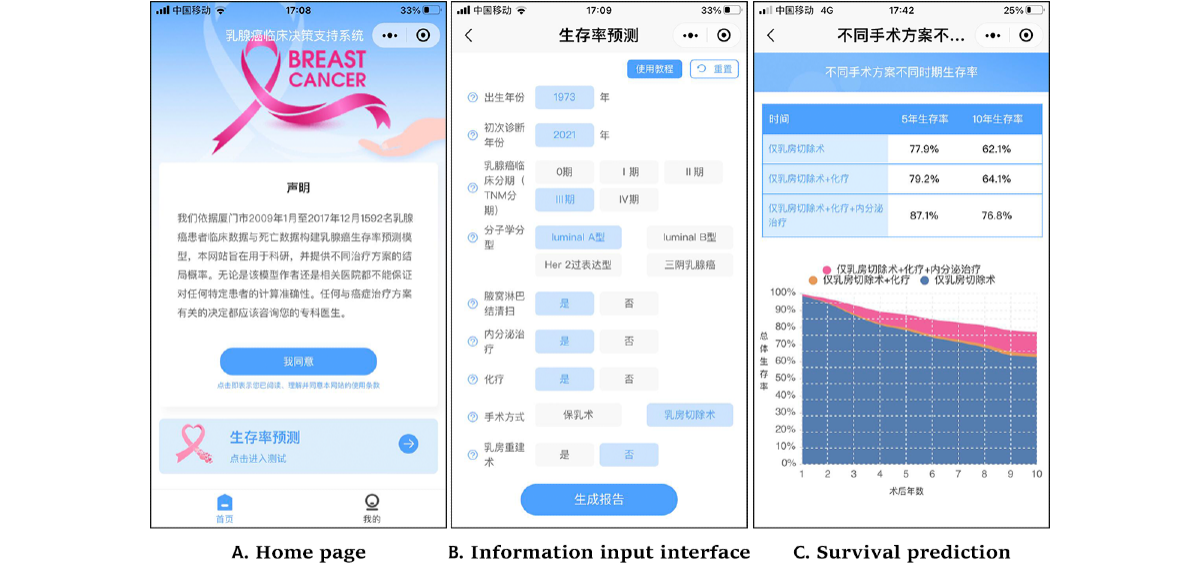
Screenshots of the iCanPredict app.

iCanPredict includes 2 function modules: survival prediction and a personal home page for women with breast cancer. The purpose and data sources are displayed on the home page with a statement as follows: “ iCanPredict aims to be used for scientific research and provide the survival probability of different treatment options. Neither the model author nor the relevant hospitals can guarantee the calculation accuracy for any specific patient. Any decision related to cancer treatment should consult your physician”. Women need to click the button “I agree” under the statement before using iCanPredict (Figure 2A).

In the survival prediction model module, there are interactive page and report page. In the interactive page, users need to input personal information, including the year of birth, the year of diagnosis, breast cancer clinical stage, and molecular classification. In addition, users need to choose surgery type (breast-conserving surgery or mastectomy), whether they will undergo reconstruction if they choose mastectomy, whether they will undergo axillary lymph node dissection, and whether they will receive chemotherapy or endocrine therapy (Figure 2B). In addition, to make it easier and faster for users to learn how to input information, there are tutorials and concept explanations beside each concept in the input information interface. Tutorials and concept explanations can resolve doubts in the use process. After the completion of information input, users need to click the button “generate report” at the bottom of the interactive page.

In the report page, iCanPredict will present the 10-year survival rates after surgery only, surgery plus chemotherapy, and surgery plus chemotherapy plus endocrine therapy based on the user’s input information. The survival rates are presented as tables and diagrams for women with breast cancer. The tables present 5- and 10-year survival rates, while the diagrams describe the continuous survival rates within 10 years after primary treatment. The predicted benefits of adjuvant chemotherapy and endocrine therapy are represented by the percentage increase in the predicted survival rate with differently colored curves in the diagram for women with breast cancer who received adjuvant therapy, compared with those who did not receive adjuvant therapy [1] (Figure 2C).

**Figure 3:**
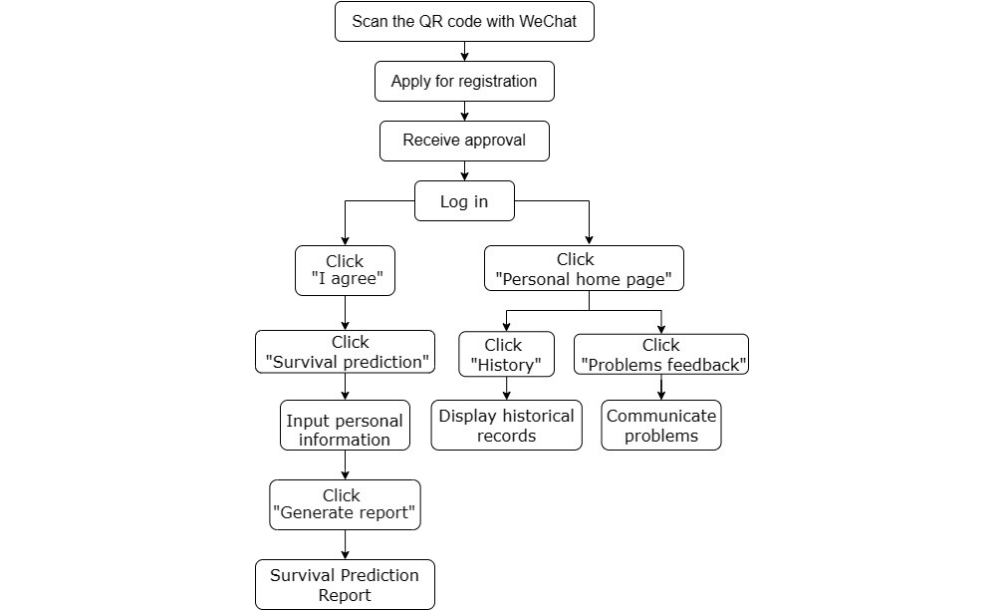
Flowchart of the use of the iCanPredict app.

Users can make different choices on different surgeries and adjuvant therapies, and the corresponding survival prediction data will be saved on their personal home page. The personal home page module includes personal centers, problems feedback, and history. Personal center refers to the account-related information (nickname, avatar, basic information, etc.), which is set up by the users themselves. Problems feedback is an interactive platform for communication between users and the iCanPredict developer group. Users can provide feedback on the problems encountered in the use of iCanPredict or provide suggestions for app improvement. History summarizes user input information and survival prediction data are expressed in tables and diagrams and arranged in chronological order for users to compare the survival rates of different surgeries and adjuvant treatments. The flowchart of the use of iCanPredict is presented in Figure 3.

A total of 6 women with breast cancer and 2 surgeons were invited to assess the perceived ease of use and perceived usefulness. Of these, 2 women were aged over 60 years and were reluctant to use the iCanPredict app. One women got confused about her own value of molecular classification and needed to refer to the laboratory test report to input information. With help from the researchers to download the iCanPredict app and explain the laboratory test, all 6 women showed great interest in the prediction model. They appraised the iCanPredict app’s convenience, ease of use, and ability to provide valuable information they wanted. The 2 surgeons indicated that it was easy to use the iCanPredict app. The surgeons expressed surprise that mastectomy and breast conservative surgery resulted in similar survival rates. They also suggested to add more information to predict survivals following other treatment options, such as immunotherapy or gene therapies. Such therapies have been emphasized to treat women with breast cancer in the recent years, but were not commonly applied before. Both women with breast cancer and surgeons indicated that it was feasible and acceptable to use the iCanPredict app in the clinics.

## Discussion

### Principal Findings

In this study, we constructed a well-calibrated prediction model on the basis of 9 years of clinical and death information for women with breast cancer in Xiamen. The survival prediction model was run in the app background thread, and the iCanPredict prognostic app was designed and developed for women with breast cancer in China.

iCanPredict was developed on the popular mobile phone platform in China (WeChat), which is easily accessible and user-friendly for both physicians and women with breast cancer [34]. iCanPredict provides individual survival predictions and calculates the benefits of the corresponding treatments. The tables display the percentage of predicted survival rates for physicians and women to compare the predicted results among different surgeries and adjuvant therapies. The use of colored diagrams to display the improvement in the predicted survival rate will facilitate sophisticated and difficult discussions on corresponding adjuvant therapies for patients [1].

### Comparison With Prior Work

In our prediction model, the type of surgery was not an independent prognostic factor for breast cancer overall survival, which is consistent with some previous studies [16,17]. When physicians discuss the choices of breast surgeries with women, they may input women’s clinical data and attempt different types of surgery in the iCanPredict app, which presents the survival prediction rate for different surgeries in an easy-to-understand manner. In combination with value preferences, women may thus make better informed decisions on surgeries [35,36].

In our prediction model, endocrine therapy was a significant prognostic factor for overall survival, which was also supported by the literature [37]. iCanPredict will present the benefit of endocrine therapy based on the percentage of improvement in the predicted survival rate if physicians or women choose endocrine therapy. iCanPredict will provide a quick, visual presentation, such as tables and diagrams, to inform women about the beneficial effects of endocrine therapy.

For clinical implementation, it is recommended that women with breast cancer use the iCanPredict together with surgeons when making decisions on treatments. Further, cautions should be exercised when using this iCanPredict app to guide the decision making, considering new therapies that are constantly emerging in recent years such as immunotherapy [38-41] or gene-based therapies [42,43], which may affect the survival rate of women with breast cancer.

### Strengths and Limitations

iCanPredict is the first tool of its kind in China to provide survival predictions to women scheduled to undergo breast surgeries and corresponding adjuvant therapies. iCanPredict was proved to have good model discrimination and calibration. This study had several limitations. First, the sample size was relatively small to build a prediction model, as there were only limited number of eligible cases in the 2 participating hospitals, which may limit the generalization of the iCanPredict app. Second, death data from the Xiamen Center CDC did not include specific causes of death; therefore, this study did not have specific breast cancer survival rates, and only contained overall survival rates. Therefore, the iCanPredict app may overestimate the mortality of breast cancer. Third, due to the incomplete clinical records of adjuvant endocrine therapy and adjuvant chemotherapy treatment, we can only predict the benefits of adjuvant therapy by determining whether adjuvant therapy was administered (yes vs no). In future studies, the adherence to adjuvant endocrine therapy should be emphasized to help clinicians and patients make accurate and individual medical decisions.

### Future Directions

Future studies need to combine the results of this study with the financial burden of different treatments [44] and patients’ own preferences [35,36] to provide better clinical decision support. Studies with large sample size and more comprehensive clinical data, in combination with the medical costs related to medical treatments and patients’ value preference, are warranted to be conducted in different countries and cultures.

### Conclusions

The prediction model had good model discrimination and model calibration to predict the overall survival of women with breast cancer in China. iCanPredict will increase women’s awareness of the similar survival rate of different surgeries and the importance of adherence to endocrine therapy, ultimately helping them make informed decisions about treatment for breast cancer. In the long run, better choice of surgery and increased adherence to prescribed endocrine therapy may be attained.

## Abbreviations

AUC: area under the curve
HR: hazard ratio
PH: proportional hazards
ROC: receiver operating characteristic
Xiamen Center CDC: Xiamen Center of Disease Control and Prevention
